# Lower brain-derived neurotrophic factor levels are associated with age-related memory impairment in community-dwelling older adults: the Sefuri study

**DOI:** 10.1038/s41598-020-73576-1

**Published:** 2020-10-05

**Authors:** Yoshito Mizoguchi, Hiroshi Yao, Yoshiomi Imamura, Manabu Hashimoto, Akira Monji

**Affiliations:** 1 Department of Psychiatry, Faculty of Medicine, Saga University, 5-1-1 Nabeshima, Saga, 849-8501 Japan; 2 Division of Clinical Research, National Hospital Organization Hizen Psychiatric Center, Saga, 842-0192 Japan

**Keywords:** Neuroscience, Psychiatric disorders

## Abstract

The beneficial effects of brain-derived neurotrophic factor (BDNF)—a member of the neurotrophin family—on cognitive function or dementia are well established in both rodents and human beings. In contrast, little is known about the association of proBDNF—a precursor protein with opposing neuronal effects of BDNF—with cognitive function in non-demented older adults. We analyzed brain magnetic resonance imaging findings of 256 community-dwelling older adults (mean age of 68.4 years). Serum BDNF and proBDNF levels were measured by quantitative enzyme-linked immunosorbent assay. Logistic regression analysis revealed that older age, less physical activity, hippocampal atrophy, and lower BDNF levels were independently associated with memory impairment determined by the Rivermead Behavioral Memory Test. Path analysis based on structural equation modeling indicated that age, sport activity, hippocampal atrophy and BDNF but not proBDNF were individually associated with Rivermead Behavioral Memory Test scores. These findings suggest that impaired BDNF function, in addition to physical inactivity and hippocampal atrophy, is associated with age-related memory impairment. Therefore, BDNF may be a potential target for dementia prevention.

## Introduction

Approximately 46.8 million people suffer from dementia worldwide, with an accompanying total estimated global cost at 818 billion dollars. Because the pathological changes related to Alzheimer’s disease (AD) are supposed to begin from 10 to 15 years before the onset of memory decline or dementia^1^, establishment of peripheral biomarkers that enable early detection of dementia or memory decline are urgently needed^2,3^.

Brain-derived neurotrophic factor (BDNF), a member of the neurotrophin family, has various important roles in neuronal differentiation and survival, neurite outgrowth, gene expression and synaptic plasticity in the rodent brain^4–6^. BDNF is also important for memory formation in rodents^7^. Moreover, BDNF is first synthesized as a precursor proBDNF protein. ProBDNF is cleaved to mature BDNF in the intracellular endoplasmic reticulum or extracellularly by proteases such as plasmin and matrix metalloproteases^8^. Interestingly, the binding of BDNF to its cognate tropomysin related kinase B (TrkB) receptors supports neuronal survival, whereas binding of proBDNF to p75 neurotrophin receptor (NTR) leads to apoptosis, indicating that proBDNF and mature BDNF elicit opposing neuronal responses in the rodent brain^9,10^. In aged mice, the expression of proBDNF is increased in the hippocampus relative to young mice and intra-hippocampal infusions of proBDNF lead to a progressive and significant impairment of memory function^11^.

In rodents, BDNF can cross the blood–brain barrier (BBB)^12^ and serum levels of BDNF are shown to well correlate with brain-tissue BDNF levels^13^. Although some reports showed serum levels of BDNF did not correlate with CSF levels of BDNF in AD patients^14^, BDNF is supposed to be one of peripheral biomarkers that enable early detection of dementia or memory decline^15^. In humans, accumulating evidence shows that hypofunction of BDNF signaling is associated with neurodegenerative diseases, including Parkinson's disease^16^ and AD^17–19^. However, it remains unclear how both proBDNF and BDNF are involved in memory decline and/or the onset of dementia among older adults^17^. To our knowledge, this is the first report to examine whether peripheral levels of both BDNF and proBDNF are associated with memory function in older adults with or without dementia. Our research might be useful for testing the hypothesis that proBDNF and mature BDNF elicit opposing roles on memory function in older adults.

## Results

### Background characteristics

**Figure 1:**
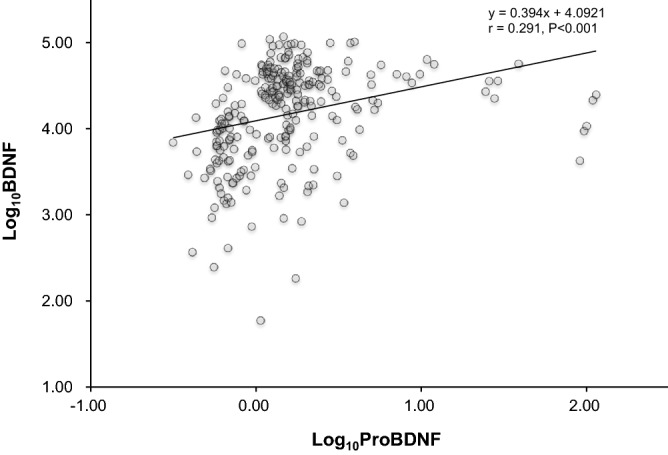
Log10 proBDNF and log10 BDNF correlated well (r = 0.290, *p* < 0.001) in our populations.

Because the distribution in levels of BDNF and proBDNF were highly skewed, the log-transformed BDNF (log_10_ BDNF) and proBDNF (log_10_ proBDNF) values were used for statistical analysis. Log_10_ proBDNF and log_10_ BDNF correlated well (r = 0.290, *p* < 0.001, Fig. 1) in the same populations. The characteristics of the study population among tertiles of BDNF values are provided in Table 1. The lowest tertile of BDNF was associated with higher age, fewer cases of metabolic syndrome, lower levels of RBMT score, diastolic blood pressure, albumin, Hemoglobin A1c, low density lipoprotein, and estimated glomerular filtration rate.

**Table 1:** Characteristics of the study population.

	BDNF tertiles			*p* for trend
	Low (n = 85)	Medium (n = 86)	High (n = 85)	
	60–9704 μg/L	9715–32,501 μg/L	32,687–115,640 μg/L	
Age, mean (SD), years	70.4 (8.3)	66.8 (5.8)	68.1 (6.3)	0.003
Male, n (%)	35 (41.1)	41 (47.7)	44 (51.8)	NS
Education, mean (SD), years	11.6 (2.5)	11.6 (2.0)	11.3 (2.1)	NS
Modified Stroop test, mean (SD)	18.8 (13.0)	18.2 (12.8)	18.1 (10.2)	NS
Rivermead Behavioral Memory Test, mean (SD)	18.5 (4.1)	19.4 (3.9)	20.0 (3.6)	0.033
Apathy scale, mean (SD)	453 (115)	470 (107)	471 (116)	NS
Body mass index, mean (SD), kg/m^2^	23.2 (3.2)	23.8 (3.7)	23.5 (3.6)	NS
Hypertension, n (%)	37 (43.5)	34 (39.5)	32 (37.6)	NS
Systolic BP, mean (SD), mmHg	142.8 (21.2)	142.6 (19.1)	139.4 (16.4)	NS
Diastolic BP, mean (SD), mmHg	78.4 (11.2)	83.3 (10.7)	84.5 (9.4)	< 0.001
Diabetes mellitus, n (%)	10 (11.8)	16 (18.6)	12 (14.1)	NS
Hyperlipidemia, n (%)	31 (36.5)	25 (29.1)	29 (34.1)	NS
Metabolic syndrome, n (%)	5 (5.9)	18 (20.9)	11 (12.9)	0.015
Chronic kidney disease, n (%)	21 (24.7)	11 (12.8)	15 (17.6)	0.129
Alcohol, n (%)	30 (35.3)	38 (44.2)	33 (38.8)	NS
Smoking, n (%)	7 (8.2)	9 (10.5)	8 (9.4)	NS
Albumin, mean (SD), g/dL	4.31 (0.36)	4.45 (0.26)	4.49 (0.30)	< 0.001
Hemoglobin A1c, mean (SD), %	5.53 (0.46)	5.79 (0.96)	5.75 (0.65)	0.045
LDL cholesterol, mean (SD), mg/dL	116.5 (32.6)	123.2 (31.9)	129.2 (33.7)	0.042
HDL cholesterol, mean (SD), mg/dL	68.7 (17.3)	67.7 (17.1)	66.5 (16.9)	NS
Triglyceride, mean (SD), mg/dL	116.5 (77.5)	119.1 (98.3)	122.6 (90.9)	NS
eGFR, mean (SD), mL/min/1.73 m^2^	70.2 (15.2)	76.8 (14.4)	75.1 (14.7)	0.012

*BP* blood pressure, *LDL* low density lipoprotein, *HDL* high density lipoprotein, *eGFR* estimated glomerular filtration rate.

NS, *p* > 0.2.

**Figure 2:**
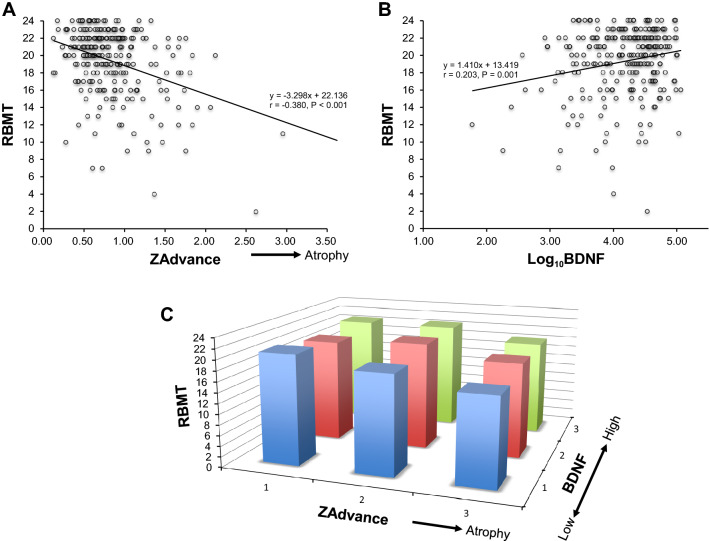
Hippocampal atrophy and lower BDNF levels were independently associated with memory impairment determined by the Rivermead Behavioral Memory Test (RBMT). (**A**) The RBMT score was negatively correlated with hippocampal atrophy (i.e., higher ZAdvance score) (Pearson correlation coefficient r = 0.380, *p* < 0.001). (**B**) The RBMT score was positively correlated with BDNF (r = 0.203, *p* = 0.001). (**C**) Hippocampal atrophy and low BDNF levels were synergistically correlated with memory dysfunction. The different colors—blue, red, and green—were used to indicate the groups with low, medium, and high tertiles of BDNF levels, respectively.

The RBMT score was negatively correlated with hippocampal atrophy (i.e., higher ZAdvance score) (Pearson correlation coefficient r = 0.380, *p* < 0.001), while the RBMT score was positively correlated with BDNF (r = 0.203, *p* = 0.001) (Fig. 2A, B). Hippocampal atrophy and low BDNF levels were synergistically correlated with memory dysfunction (Fig. 2C).

### Logistic regression analysis

Multivariate analysis was carried out with logistic regression analysis for cognitive function as the dependent variable and the log_10_ BDNF value, age, sex, education, physical activity, sport index, and MRI findings as the independent variables. When possible confounders were entered into the binary logistic regression model (the forward stepwise method), the independent predictors of memory dysfunction (RBMT < 17) were age (OR = 2.575/10 years; 95% CI 1.540–4.305; *p* < 0.001), log_10_ BDNF (OR = 0.546; 95% CI 0.299–0.999; *p* = 0.050), sport activity (OR = 0.346; 95% CI 0.134–0.895; *p* = 0.029), and hippocampal atrophy (OR = 0.546/z score; 95% CI 0.299–0.999; *p* = 0.050); MRI findings such as silent brain infarction, white matter lesions, and cerebral microbleeds did not enter into the equation via the forward stepwise procedure, while the independent predictors of executive dysfunction (modified Stroop test ≥ 27 s) were age (OR = 4.272; 95% CI 2.604–7.009; *p* < 0.001), and sport activity (OR = 0.391; 95% CI 0.154–0.989; *p* = 0.047) (Table 2).

**Table 2:** Potential correlating factors for cognitive function.

	Rivermead Behavioral Memory Test			Modified Stroop test		
	OR	95% CI	*p*	OR	95% CI	*p*
Age, /10 years	2.575	1.540–4.305	0.000	4.272	2.604–7.009	0.000
Log_10_BDNF	0.546	0.299–0.999	0.050			
Sport*	0.346	0.134–0.895	0.029	0.391	0.154–0.989	0.047
ZAdvance	4.489	1.907–10.564	0.001			

Age, sex, education, physical acitivity, sport index, log_10_BDNF, and MRI findings were included in the forward stepwise method of logistic regression analysis.

*Sport activity was defined as sport index 4.0 MET × hour/week or over.

### Structural equation modeling

**Figure 3:**
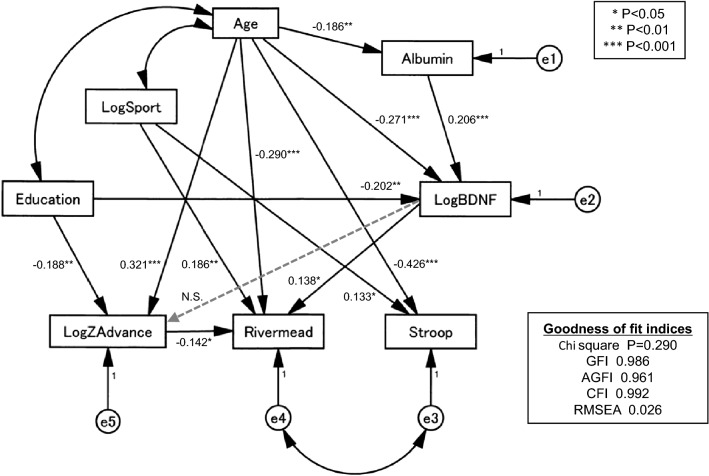
Path analysis showing that age, sport activity, hippocampal atrophy and BDNF were individually associated with Rivermead Behavioral Memory Test scores. Path analysis based on structural equation modeling indicated that the direct paths from age (β =  −0.321, *p* < 0.001), sport (β = 0.191, *p* < 0.001), hippocampal atrophy (ZAdvance) (β =  −0.220, *p* < 0.001), and log10 BDNF (β =  −0.150, *p* < 0.01) to memory dysfunction (Rivermead) were significant.

The findings mentioned above led us to the hypothesis that age, sport activity, hippocampal atrophy and lower BDNF levels caused memory dysfunction, while hippocampal atrophy and low BDNF levels were not associated with executive dysfunction. We investigated the relationship between cognitive function tests, hippocampal atrophy, sport activity, and the log_10_ BDNF value, using a graphical multivariate analysis SEM. Path analysis based on SEM indicated that the direct paths from age (β = 0.290, *p* < 0.001), sport (β = 0.186, *p* = 0.001), hippocampal atrophy (ZAdvance) (β = 0.142, *p* = 0.019), and log_10_ BDNF (β = 0.138, *p* = 0.015) to memory dysfunction (Rivermead) were significant (Fig. 3). Although the direct path from age to executive dysfunction (Stroop) were significant (β =  − 0.426, *p* < 0.001), the direct paths from log_10_ BDNF to hippocampal atrophy and executive dysfunction were not significant. These results were quite similar to those from the logistic regression analysis shown in Table 2. The direct path from log_10_ BDNF to hippocampal atrophy was not significant, while the direct paths from age and education to hippocampal atrophy were significant (β = 0.321, *p* < 0.001 and β =  − 0.188, *p* = 0.004, respectively), as had been shown in our previous study^20^. The measures of model fitness were as follows: chi-square N.S., GFI = 0.986, AGFI = 0.961, CFI = 0.992, and RMSEA = 0.026. Thus, the presented model reasonably fit the data.

## Discussion

In the present cross-sectional study, logistic regression analysis revealed that age, sport activity, hippocampal atrophy, and BDNF were independently associated with memory function determined by RBMT. Path analysis based on SEM indicated that age, sport activity, hippocampal atrophy and BDNF but not proBDNF were individually associated with RBMT. These findings suggested that physical inactivity, hippocampal atrophy and hypofunction of BDNF were individually associated with age-related memory impairment; these may all be strategic targets for dementia prevention.

To our knowledge, this is the first report to examine whether peripheral levels of both BDNF and proBDNF are associated with memory function in older adults without dementia.

ProBDNF modulates synaptic plasticity, including the induction or enhancement of long-term depression^21^, through the inhibition of dendritic outgrowth in rodent hippocampus^22^. In addition, proBDNF increases the amount of amyloid β (Aβ) deposition and induces memory dysfunction in mice models of AD^23^. In this study, however, SEM revealed that proBDNF was not directly associated with memory dysfunction in community-dwelling older adults. BDNF modulates synaptic plasticity, including the induction or enhancement of long-term potentiation^24^, through the promotion of neurogenesis^25^ and/or dendritic outgrowth^26,27^ in rodent hippocampus. These findings are consistent with our observation showing that hypofunction of BDNF was associated with age-related memory impairment. In addition, exercise enhances memory function in both rodents^28^ and human^29^. These effects are accompanied by increased neuronal proliferation or survival and enhancement of dendritic outgrowth in the hippocampus^30^, mainly mediated by BDNF signaling^31,32^. Thus, we could not dissociate the potential effects of lower BDNF and lower physical exercise on memory impairments in the present study. In healthy young college students, exercise (a short period of high-intensity cycling) results in the enhancement of hippocampus-related memory function (face–name matching) without affecting the Stroop word–color test, which was accompanied by increased serum BDNF levels^33^. Likewise, Frerris et al. reported that exercise (graded exercise test) improved the Stroop word–color test, but they could not find any significant correlation between the improvement of the Stroop test and an increase in BDNF^34^. These reports are compatible with our findings that BDNF was associated with RBMT but not the Stroop test. In contrast, Giacobbo et al. found a significant correlation between scores of the Stroop test and serum concentration of BDNF in young adults who were deprived of sleep^35^. The Stroop word–color test is shown to recruit the anterior cingulate cortex and other frontal cortical regions^36^. We need further investigations with a larger sample size to clarify the relationship between BDNF and the Stroop test in older adults.

Apathy is supposed to be a risk factor^37^ or prodromal symptom^38^ of AD. Alvarez et al. have reported that apathy is associated with lower levels of BDNF in AD patients; in that study, apathy was evaluated using the Neuropsychiatric Inventory^39^. In the present study, however, SEM analysis revealed that both BDNF and proBDNF were not associated with the apathy scale in community-dwelling older adults (unpublished observation). These discrepancies might be due to differences in the evaluation methods for apathy; we used Starkstein’s apathy scale in this study. In addition, we recently reported that low-grade inflammation was associated with apathy indirectly via confluent DWMLs^40^. Thus, low-grade inflammation rather than BDNF signaling might be associated with apathy in older adults.

We noticed some limitations 1) cross-sectional study design limited our interpretation of results regarding the cause and effect and 2) we could not exclude the possible residual confounding factors related to the SEM analysis. In addition, we used maximum likelihood estimation method by converting values of sport activity and ZAdvance to logarithm and by dividing scores of Rivermead and Stroop tests to quintile. As a result, we could reach multivariate kurtosis value to 4.143, although it did not mean normality (< 1), but moderate non-normality (< 10) by excluding proBDNF from the SEM analysis. Although the strength of our study is its measurements of both proBDNF and BDNF in each subject, the newly identified BDNF pro-peptide—proBDNF is cleaved to BDNF and this BDNF pro-peptide^41^—was not measured, leaving the possibility that BDNF pro-peptide could interfere with the statistical effects of proBDNF on memory function.

In conclusion, we suggest that physical inactivity, hippocampal atrophy and hypofunction of BDNF signaling individually associate with age-related memory dysfunction and they might all be targets to prevent dementia.

## Methods

### Participants and protocol approval

Between 2010 and 2016, we performed a cross-sectional observational study in the rural community of Sefuri village (Saga, Japan), which had a total population of 1739 people as of April 2014^40^. We examined 297 consecutive volunteers aged 60–89 years, who were independent in their daily life without apparent dementia. Subjects apparently not eligible for this study were excluded; these were cognitive impairment (*n* = 7); psychiatric disorders, including depression (*n* = 5); claustrophobia or contraindications for MRI (*n* = 8); a history of stroke (*n* = 9); brain tumor (*n* = 1); chronic subdural hematoma (*n* = 1); a history of head trauma (*n* = 3); chronic renal failure (*n* = 3); and insufficient clinical information (*n* = 4). Finally, we analyzed 256 subjects in the present study. This study was approved by the National Hospital Organization Hizen Psychiatric Center Institutional Review Board approved the study (approval numbers: 15–1 and 24–4) and procedures were carried out in accordance with the approved guidelines. Written informed consent was obtained from all participants.

### Clinical assessments

The participants underwent a structured clinical interview, general hematology and biochemical tests. Blood pressure was measured in the sitting position using the standard cuff method; beginning in 2013, simultaneous blood pressure measurements were recorded from both arms using a pair of automated sphygmomanometers (Omron model HEM-1020, Omron, Japan). Vascular risk factors were defined as previously described^40^. Briefly, arterial hypertension was considered to be present in participants with a history of repeated blood pressure recordings ≥ 140/90 mmHg, and in those being treated for hypertension. Diabetes mellitus was defined as a fasting plasma glucose level of ≥ 6.99 mmol/L (126 mg/dL) and/or HbA1c of ≥ 6.5%, or a previous diagnosis of diabetes mellitus. Hyperlipidemia was considered to be present in participants with a total serum cholesterol concentration of ≥ 5.69 mmol/L (220 mg/dL), and in those being treated for hyperlipidemia. Metabolic syndrome was defined by the presence of central obesity and a minimum of two of three factors: a blood pressure of ≥ 130/85 mmHg, a fasting blood glucose level of ≥ 6.1 mmol/L (110 mg/dL), and a triglyceride level of ≥ 1.69 mmol/L (150 mg/dL) and/or HDL cholesterol level of < 1.03 mmol/L (40 mg/dL). The estimated glomerular filtration rate (eGFR) was calculated using the Modification of Diet in Renal Disease equation for the Japanese modification as previously described^37^. Smoking was defined as the participant smoking an average of at least 10 cigarettes per day, while former smokers were considered nonsmokers. Alcohol use was defined as the participant reporting drinking one or more alcoholic beverages (10 g of ethanol) per week.

### Physical activity

Physical activity was assessed with a questionnaire modified from the Baecke questionnaire on habitual physical activity^42^. The questionnaire consisted of three components: leisure time, work, and sport activities. Items concerning leisure time and work activities were coded on five-point scales. The leisure time index was assessed with four questions on comparison with others, sweat, sport, and walking. The work index was assessed through four questions on standing, walking, heavy loads, and sweat. The sport index was expressed as reported hours per week in each category multiplied by the metabolic equivalent of task (MET)^43^. Sport activity was defined as sport index 4.0 MET× hours/week or over.

### Assessment of cognitive function

Participants were tested individually in a quiet room. All participants underwent the Rivermead Behavioral Memory Test (RBMT) for memory function assessment and the modified Stroop test for executive or frontal lobe function assessment as previously described^20,44^. Briefly, the RBMT consists of 11 subtests—first and second names, belonging, appointment, picture recognition, story (immediate, delay), face recognition, route (immediate, delay), messages (immediate, delay), orientation and date—with four parallel forms designed to identify memory deficits that might be encountered during daily living^45^. The test does not adhere to any particular theoretical model of memory; instead, it attempts to mimic the demands made on memory by normal daily life. The standard profile score of RBMT yields scores between 0 and 24 (higher scores indicate better performance); subjects with a standard profile score of RBMT less than 17 (the lowest quintile) were operationally defined as having memory impairment as previously described^20^. The modified Stroop test comprises two parts; subjects were asked to name the colors of dots in Part I and colors of incongruent words (Chinese characters) in Part II. The difference in time between the two parts was considered to be due to interference effects, and the subject was considered to have executive dysfunction if the total score was above the most prolonged fifth quintile (i.e., ≥ 27 s in the present study).

### Apathy scale

Each item of the Starkstein apathy scale^46^ was quantified on a visual analog scale, where one end of a 60-mm long line is ‘absolutely correct’ and the other end is ‘completely wrong’, as previously described^40,47,48^. Because the item–total correlations of questions 3 (Are you concerned about your condition?) and 11 (Are you unconcerned with many things?) had been the weakest of the 14 original questions, we excluded the scores of these two questions from the analysis. This apathy scale yields total scores of 0–720, with lower scores indicating apathetic behavior. Depressed mood and insomnia were rated as ‘none’, ‘sometimes’, ‘frequent’, and ‘always’; depressed mood and insomnia were defined as an always or frequent presence of these symptoms. Patients who had been previously diagnosed with clinical depression or were taking medication for depression were excluded from the study.

### Measurement of serum proBDNF and BDNF levels

Serum specimens were stored at − 80 °C until the measurements were performed. Serum levels of proBDNF and BDNF were measured using the human proBDNF enzyme-linked immunosorbent assay (ELISA) Kit (Adipo Bioscience, Santa Clara, CA, USA) and the human BDNF ELISA Kit (Adipo Bioscience), respectively. We selected these ELISA kits because some human BDNF ELISA kits frequently used in previous reports recognized not only BDNF but also proBDNF because of limited specificity^49^. To minimize assay variance, serum levels of proBDNF and mature BDNF from each subject were measured on the same day. All samples were analyzed in duplicate. Measurements were performed according to the manufacturer's instructions and based on our experiences as previously reported^50–53^.

### Assessment of MRI findings

A combination of T1-weighted, T2-weighted, and fluid attenuated inversion recovery images (FLAIR) is required to accurately detect both silent brain infarction and white matter lesions^54^. Imaging was performed on a 1.5 T MRI scanner (Achieva, Philips, the Netherlands) using the T1- and T2-weighted, fluid-attenuated inversion recovery, and T2*-weighted images. Silent brain infarction was defined by low signal intensities on T1-weighted images, and high signal intensity areas on T2-weighted images, and a diameter of ≥ 3 mm, as previously described. We differentiated enlarged perivascular spaces from silent brain infarction based on their location, shape, and size. Lesions of < 3 mm in diameter are more likely to be perivascular space than lacunes, and the presence of moderate to severe basal ganglia perivascular space was recorded^55^. The white matter lesions were defined as isointense with normal brain parenchyma on T1-weighted images, and high signal intensity areas on T2-weighted images. We used the validated rating scale of DWMLs by Fazekas et al.: grade 0, absent; grade 1, punctate foci; grade 2, beginning confluence of foci; and grade 3, large confluent areas^56^. For periventricular hyperintensities, we determined the presence and severity (grade 0, absent; grade 1, pencil thin; and grade 2, smooth halo lining) using FLAIR images. Two researchers who were blinded to all clinical data, independently reviewed all scans. We evaluated the degree of hippocampal atrophy, using a free software program—the Voxel-based Specific Regional analysis system for Alzheimer’s Disease (VSRAD) advance version based on statistical parametric mapping 8 (SPM8) plus Diffeomorphic Anatomical Registration Through an Exponentiated Lie algebra (DARTEL)^57^. To preserve gray matter volume within each voxel, Matsuda et al. modulated the images by the Jacobean determinants derived from the spatial normalization by DARTEL. We checked the segmentation process according to the VSRAD manual (https://www.vsrad.info/index2.html); a qualified technician (K. Kawakami) confirmed good contrast between gray and white matter, no unacceptable irregularity in the images, no apparent artifacts, no unacceptable low intensities in T1-weighted images, and no unacceptable ventricular enlargements. We determined the extent of atrophy as the averaged value of positive voxel-by-voxel z-scores, where z-score = ([control mean] − [individual value]) / (control SD) (i.e., the higher the value, the higher the extent of atrophy). We used three indicators—the severity of atrophy obtained from the averaged positive values of *z*-score in the target (hippocampus) volume of interest (VOI) (hereafter referred to as ZAdvance), and the percentage rates of the coordinates with the *z*-score exceeding the threshold value of 2.00 in the target (hippocampus) VOI and in the whole brain VOI—for characterizing atrophy of the hippocampus and the whole brain. Although the normative data set was an external one created by Matsuda et al.^57^, age and sex were well balanced between our subjects and the external normative data set (i.e., 37 men and 43 women with a mean age of 70.4 ± 7.8 (SD) years; we examined 256 subjects (120 men and 136 women) with a mean age of 68.4 ± 7.0 (SD) years with a compatible MRI protocol.

### Statistical analysis

All clinical variables are presented as the mean ± standard deviation. All tests were two-sided, and the level of statistical significance was set at *p* < 0.05. The data were analyzed using IBM SPSS Statistics version 18 for Windows (SPSS Japan Inc., Tokyo, Japan). For the univariate analysis, the chi-square test or Fisher’s exact test were used to investigate between-group differences in categorical variables, while unpaired t-tests were used to investigate differences in continuous variables. Pearson’s correlation coefficients were used to assess the relationship between the log_10_ BDNF value or hippocampal atrophy and RBMT score. Multiple comparisons were performed using ANOVA, followed by Bonferroni testing. Multivariate analysis was carried out with the forward stepwise method of logistic regression analysis. The association between the cognitive function tests and the BDNF values was tested using binary logistic regression analysis, adjusted for age, sex, education, physical activity, sport index, and MRI findings. We investigated the relationship between cognitive function tests, age, education, physical activity, sport index, hippocampal atrophy, and BDNF using structural equation modeling (SEM)^58^. We performed SEM analysis on 256 subjects, excluding three subjects whose proBDNF levels exceeded the maximum concentration measurable by ELISA. The SEM was described as path diagrams, wherein the square boxes represented measured observations and circles represented latent constructs. Single-headed arrows represented a simple regression relationship and double-headed arrows represented correlations. The parameters estimation was done with maximum likelihood estimation (the default setting of AMOS). To construct the SEM model, we investigated the variables associated with memory dysfunction, and logistic regression analysis revealed that older age, less sport activity, hippocampal atrophy, and lower BDNF levels were independently associated with memory impairment determined with the RBMT. Executive function determined with the Stroop test was added to this model, because executive function is a distinctive ability from memory. Consequently, model fit for SEM analysis did not lead to an inflated chi-square test value. We also examined several indices of model fit for SEM analysis with their acceptable thresholds: low chi-square values relative to degrees of freedom with an insignificant P value (*p* > 0.05); values > 0.95 for goodness of fit index (GFI), adjusted goodness of fit index (AGFI), and comparative fit index (CFI); values < 0.07 for root mean square error of approximation (RMSEA).

## References

[1] McDade E, Bateman RJ (2017). Stop Alzheimer's before it starts. Nature.

[2] Nakamura A (2018). High performance plasma amyloid-β biomarkers for Alzheimer's disease. Nature.

[3] Hampel H (2018). Blood-based biomarkers for Alzheimer disease: Mapping the road to the clinic. Nat. Rev. Neurol..

[4] Mizoguchi Y, Ishibashi H, Nabekura J (2003). The action of BDNF on GABA(A) currents changes from potentiating to suppressing during maturation of rat hippocampal CA1 pyramidal neurons. J. Physiol..

[5] Park H, Poo MM (2013). Neurotrophin regulation of neural circuit development and function. Nat. Rev. Neurosci..

[6] Vacher M (2019). Validation of a priori candidate Alzheimer's disease SNPs with brain amyloid-beta deposition. Sci. Rep..

[7] Bekinschtein P, Cammarota M, Medina JH (2014). BDNF and memory processing. Neuropharmacology.

[8] Pang PT (2004). Cleavage of proBDNF by tPA/plasmin is essential for long-term hippocampal plasticity. Science.

[9] Greenberg ME, Xu B, Lu B, Hempstead BL (2009). New insights in the biology of BDNF synthesis and release: implications in CNS function. J. Neurosci..

[10] Mizoguchi Y, Monji A (2017). Microglial intracellular Ca^2+^ signaling in synaptic development and its alterations in neurodevelopmental disorders. Front. Cell. Neurosci..

[11] Buhusi M, Etheredge C, Granholm AC, Buhusi CV (2017). Increased hippocampal ProBDNF contributes to memory impairments in aged mice. Front. Aging Neurosci..

[12] Pan W (1998). Transport of brain-derived neurotrophic factor across the blood-brain barrier. Neuropharmacology.

[13] Klein AB (2011). Blood BDNF concentrations reflect brain-tissue BDNF levels across species. Int. J. Neuropsychopharmacol..

[14] Laske C (2007). BDNF serum and CSF concentrations in Alzheimer's disease, normal pressure hydrocephalus and healthy controls. J. Psychiatr. Res..

[15] Ng TKS (2019). Decreased serum brain-derived neurotrophic factor (BDNF) levels in patients with Alzheimer's disease (AD): A Systematic Review and Meta-Analysis. Int. J. Mol. Sci..

[16] Palasz E (2020). BDNF as a promising therapeutic agent in Parkinson's disease. Int. J. Mol. Sci..

[17] Lu B (2013). BDNF-based synaptic repair as a disease-modifying strategy for neurodegenerative diseases. Nat. Rev. Neurosci..

[18] Weinstein G (2014). Serum brain-derived neurotrophic factor and the risk for dementia: The Framingham Heart Study. JAMA Neurol..

[19] Salinas J (2017). Associations between social relationship measures, serum brain-derived neurotrophic factor, and risk of stroke and dementia. Alzheimers Dement. NY.

[20] Hashimoto M (2016). Hippocampal atrophy and memory dysfunction associated with physical inactivity in community-dwelling elderly subjects: The Sefuri study. Brain Behav..

[21] Woo NH (2005). Activation of p75NTR by proBDNF facilitates hippocampal long-term depression. Nat. Neurosci..

[22] Yang J (2014). proBDNF negatively regulates neuronal remodeling, synaptic transmission, and synaptic plasticity in hippocampus. Cell Rep..

[23] Chen J (2017). proBDNF accelerates brain amyloid-β deposition and learning and memory impairment in APPswePS1dE9 transgenic mice. J. Alzheimers Dis..

[24] Figurov A (1996). Regulation of synaptic responses to high-frequency stimulation and LTP by neurotrophins in the hippocampus. Nature.

[25] Choi SH (2018). Combined adult neurogenesis and BDNF mimic exercise effects on cognition in an Alzheimer's mouse model. Science.

[26] Horch HW, Katz LC (2002). BDNF release from single cells elicits local dendritic growth in nearby neurons. Nat. Neurosci..

[27] Lu Y, Christian K, Lu B (2008). BDNF: A key regulator for protein synthesis-dependent LTP and long-term memory?. Neurobiol. Learn. Mem..

[28] Di Loreto S (2014). Regular and moderate exercise initiated in middle age prevents age-related amyloidogenesis and preserves synaptic and neuroprotective signaling in mouse brain cortex. Exp. Gerontol..

[29] Erickson KI (2011). Exercise training increases size of hippocampus and improves memory. Proc. Natl. Acad. Sci. USA.

[30] van Praag H (2005). Exercise enhances learning and hippocampal neurogenesis in aged mice. J. Neurosci..

[31] Sartori CR (2011). The antidepressive effect of the physical exercise correlates with increased levels of mature BDNF, and proBDNF proteolytic cleavage-related genes, p11 and tPA. Neuroscience.

[32] Leckie RL (2014). BDNF mediates improvements in executive function following a 1-year exercise intervention. Front. Hum. Neurosci..

[33] Griffin ÉW (2011). Aerobic exercise improves hippocampal function and increases BDNF in the serum of young adult males. Physiol. Behav..

[34] Ferris LT, Williams JS, Shen CL (2007). The effect of acute exercise on serum brain-derived neurotrophic factor levels and cognitive function. Med. Sci. Sports Exerc..

[35] Giacobbo BL (2016). Could BDNF be involved in compensatory mechanisms to maintain cognitive performance despite acute sleep deprivation? An exploratory study. Int. J. Psychophysiol..

[36] Leung HC (2000). An event-related functional MRI study of the stroop color word interference task. Cereb. Cortex.

[37] van Dalen JW (2018). Association of apathy with risk of incident dementia: A systematic review and meta-analysis. JAMA Psychiatry.

[38] Mori T (2014). Apathy correlates with prefrontal amyloid β deposition in Alzheimer's disease. J. Neurol. Neurosurg. Psychiatry.

[39] Alvarez A (2014). Apathy and APOE4 are associated with reduced BDNF levels in Alzheimer's disease. J. Alzheimers Dis..

[40] Yao H (2019). Low-grade inflammation is associated with apathy indirectly via deep white matter lesions in community-dwelling older adults: The Sefuri study. Int. J. Mol. Sci..

[41] Mizui T (2019). Cerebrospinal fluid BDNF pro-peptide levels in major depressive disorder and schizophrenia. J. Psychiatr. Res..

[42] Baecke JA, Burema J, Frijters JE (1982). A short questionnaire for the measurement of habitual physical activity in epidemiological studies. Am. J. Clin. Nutr..

[43] Ainsworth, B.E. et al. *The Compendium of Physical Activities Tracking Guide*. Healthy Lifestyles Research Center, College of Nursing & Health Innovation, Arizona State University. Retrieved [Dec 17, 2014] from the World Wide Web. https://sites.google.com/site/compendiumofphysicalactivities/

[44] Yao H (2017). Chronic kidney disease and subclinical brain infarction increase the risk of vascular cognitive impairment: The Sefuri study. J. Stroke Cerebrovasc. Dis..

[45] Wilson B, Cockburn J, Baddeley A, Hiorns R (1989). The development and validation of a test battery for detecting and monitoring everyday memory problems. J. Clin. Exp. Neuropsychol..

[46] Starkstein SE (1992). Reliability, validity, and clinical correlates of apathy in Parkinson's disease. J. Neuropsychiatry Clin. Neurosci..

[47] Yao H (2009). Hypertension and white matter lesions are independently associated with apathetic behavior in healthy elderly subjects: the Sefuri brain MRI study. Hypertens. Res..

[48] Yao H (2015). Leisure-time physical inactivity associated with vascular depression or apathy in community-dwelling elderly subjects: The Sefuri study. J. Stroke Cerebrovasc. Dis..

[49] Yoshida T (2012). Decreased serum levels of mature brain-derived neurotrophic factor (BDNF), but not its precursor proBDNF, in patients with major depressive disorder. PLoS ONE.

[50] Mizoguchi Y (2012). The effect of oral presentation on salivary 3-methoxy-4-hydroxy-phenylglycol (MHPG) and cortisol concentrations in training doctors: A preliminary study. Endocrine.

[51] Nabeta H (2014). Association of salivary cortisol levels and later depressive state in elderly people living in a rural community: A 3-year follow-up study. J. Affect. Disord..

[52] Imamura Y (2017). An association between belief in life after death and serum oxytocin in older people in rural Japan. Int. J. Geriatr. Psychiatry.

[53] Mizoguchi Y (2019). The effect of continuous positive airway pressure (CPAP) treatment on serum levels of proBDNF and mature BDNF in patients with obstructive sleep apnea. Sleep Breath..

[54] Sasaki M (2008). Discriminating between silent cerebral infarction and deep white matter hyperintensity using combinations of three types of magnetic resonance images: A multicenter observer performance study. Neuroradiology.

[55] Yakushiji Y (2014). Topography and associations of perivascular spaces in healthy adults: The Kashima scan study. Neurology.

[56] Fazekas F (1993). Pathologic correlates of incidental MRI white matter signal hyperintensities. Neurology.

[57] Matsuda H (2012). Automatic voxel-based morphometry of structural MRI by SPM8 plus diffeomorphic anatomic registration through exponentiated lie algebra improves the diagnosis of probable Alzheimer Disease. AJNR Am. J. Neuroradiol..

[58] Haenlein M, Kaplan AM (2004). A beginner’s guide to partial least squares analysis. Underst. Stat..

